# Chicago Society of Physicians and Surgeons, Meeting Sept. 13

**Published:** 1875-11

**Authors:** E. Warren Sawyer


### Reports of Societies.

### CHICAGO SOCIETY OF PHYSICIANS AND SURGEONS.

*Regular Meeting, Sept.* 13, 1875.

(Reported by E. WARREN SAWYER, M.D.)

President, Dr. Bevan, in the chair.

Prof. J. S. Jewell presented to the Society an elaborate and interesting paper on motor centres in the cerebral cortex. The entire paper should be read in order to learn the interesting manner in which the subject is analyzed, as well as the views of the author, but in our report we are limited to a very imperfect abstract.

After speaking of the importance of the subject, and stating that physiologists are divided on the question of centres in the cortex, the writer says, "we do not now so much need new experiments in many departments of physiology, as we do patient, critical reflection on those we now have."

"The opinion that the cortex of the brain contains circumscribed spots, which are more or less directly concerned in the movements of definite groups of muscles, has an experimental basis. The experiments have been of two kinds." First, mutilating a considerable portion of the cortex of some adult animal's brain, as has been done by experimenters in various ways—removal of the portion, destroying the portion by means of corrosive chemical substances, or by injecting lycopodium powder into some small vessel which is supplied to a certain region of the brain, thus destroying that region. Second, excitative experiments, in which the integrity of the part excited was not destroyed. It was this latter class of experiments that formed the especial subject of the reader's remarks: "The establishment of *one* well-defined motor centre will involve the principle as truly as would a dozen."

The two experimenters who have done most in this direction, are Hitzig, of Berlin, and Ferrier, of London, and both think they have demonstrated the presence of motor or psycho-motor centres in the brain. "Their opinion seems to be, that certain limited tracts of cells, in the cortex, constitute the primary motor centres, in which, and on which, the will acts to originate voluntary action in certain definite groups of muscles. The will communicates an impulse to the cells of these centres, which are thus excited to activity. An impulse is sent from them, down certain fibres, which stand connected with them, and this impulse is conveyed to the ganglionic motor masses at the base of the brain, and they in turn are excited by the impulse, which has reached them from the centre in the cortex, and so on until the muscle, or group of muscles, is reached, which it is necessary to put in action."

These men further suppose that these centres can be put in action artificially, as they are naturally by the will; and this has been done by passing a galvanic current through very delicate electrodes plunged into various regions of the brain. The reader exhibited plates of animal brains, showing the location of certain centres. Thus it was found that in stimulation of a certain tract, the animal being under the influence of chloroform, "the movements began by wagging the tail, and spasmodic twitchings of the left ear. After the cessation of the more violent spasms, the animal held up its head, opened its eyes wide, with the most animated expression, and wagged its tail in a fawning manner."

The views of these observers have been vigorously combatted by others upon vàrious grounds. The principal objections to these views were then taken up by the reader; the following, among others: "It has been objected to the opinion as to the existence of motor centres in the cortex of the brain, that electrical excitation of the brain, after division of the facial nerve, has led in some cases to contractions of the facial muscles. It is said that the currents must have been diffused, in such a case, so as to excite the facial nerve beyond the cut, which fact, if true, would militate against the local action of the currents employed in such experiments of Hitzig, Ferrier, and others. This particular objection is made by Dr. Austin Flint, Jr., of New York." In answer to this objection, the reader says: "In this case, if we admit that wide diffusion of the current took place, we may suppose that the peripheral end of the divided facial nerve was excited, and in this way the contractions were produced, certainly they could not have arisen from an excitation of the nucleus for the facial. But if the diffusion was so wide-spreading in its action, as it must have been in this instance, it is difficult to see why both sides of the face did not act, and indeed, why more general convulsions did not occur, instead of being so nearly limited to the facial as they were, that is, if such limitation was a fact."

The reader then detailed the objections raised by Brown-Séquard, in an article in the *Boston Medical and Surgical Journal*, July 29, 1875. The first objection of this eminent physiologist is, "that the psycho-motor centres, even if we admit their existence, are not situated in homologous parts of the brains of different animals, as they ought to be if they really existed. This objection, Dr. Brown-Séquard thinks to be fatal to the hypothesis of definite cortical centres." In answer to this, the reader said: "if this objection is well-founded, it would be a serious, but, even then, not necessarily a fatal one, so it seems to me. But I am not ready, as yet, to admit it as being well founded, until still more careful studies are made, on the one hand, in respect to equivalent convolutions in the brains of different animals, as compared together; and, on the other hand, until the location of motor centres, if they exist, shall have been made the subject of a vastly greater number of experiments, and from a more enlightened stand point, than it has been possible to occupy up to this time. * * * Especially will it become apparent, that after all that has been done by Vrolik, Reichert, Leuret, and Graliolet, Owen, Huxley, Turner, Broca, Ecker, and others, towards a comparative study of the convolutions of the brain, that, except in a rather rude way, we are in no position to declare with certainty what special convolutions, or pairs of them, are truly homologous."

It is further objected, "that the character of the symptoms in brain diseases is not in the least dependent upon the seat of the lesion; so that a lesion of the same part may produce a great variety of symptoms, while, on the other hand, the same symptoms may be due to the most various causes; various, not only as regards the kind, but also the seat of the organic alteration. In view of these facts, I have been led to believe that lesions of the brain produce symptoms, not by destroying the function of the part where they exist, but by exerting over distant parts either an inhibitory or an exciting influence; or, in other words, either by stopping an activity or by setting it in play."

To all which the reader replied: "Is it possible that the brain is an almost absolute exception in this respect to other organs of the body, and, indeed, to most other parts of the nervous system, the spinal cord, for example? Is it possible that exactly the same kind and degree of a lesion of a definite part of the brain, will give rise at one time to one class or kind of symptoms, and at another, to another kind, and so on, every time producing different phenomena, or symptoms? His statement would seem either to imply that the various parts of the brain—cortex, if you please—if it can be said to have parts, either have the most variable or miscellaneous functions, or none at all. For my own part, I cannot look upon the present unsettled state of our knowledge of the structure, functions, and diseases of the brain, in any such discouraging way. The present uncertainty which prevails in the diagnosis of many brain diseases, depends on a want, as yet, of a correct or exact knowledge of the actual structure of the brain; *i. e.*, a want of a similar knowledge of the functions of its special parts, such as a more profound study of its anatomy, I believe, will reveal in the future; and, because of the positive errors that now prevail on these subjects, that the progress of future research is destined to clear away, and especially on account of the want hitherto of thoroughness in working up the *ante*, and especially the *post*-mortem histories of brain diseases. I assert, in the most unhesitating manner, that the lists of cases, say, of cerebral hæmorrhage, which are collected by such writers, for example, as Sintrac, and on which reliance is placed by Brown-Séquard, useful as they are on many accounts, are for all purposes of exact study and reliable inductions nearly worthless." The reader quoted a case, taken at random from Sintrac's book, to show how incompletely it was reported: "Male, fifty years. May; delirium, loquacity, irregular movements of the members, (dyspnœa), death. Pia mater adherent to the brain, *at the anterior and inferior part* of the right middle lobe cortical layer, *to the extent of two convolutions*, filled with grumous blood, mixed with cerebral tissue. Subjacent medullary substance intact. Brain soft. Pleuritic exudations, nodules of pulmonary hepatization, pericarditis, softness of the heart." "Now what shall we say of conclusions based on a study of such cases as this one? Is it any wonder that if they were relied on, the observer might reach such a conclusion as Dr. Brown-Séquard has done? What part of the brain proper was diseased in the case I have quoted? Why, 'the anterior and inferior part of the right middle lobe,' and beside this, 'the brain was soft.' But the point in these remarks is this, that it is by the study of a collection of similar cases that Dr. Brown-Séquard reaches the conclusion that 'the symptoms in brain diseases are not in the least dependent on the seat of the lesion;' and conclusions based on a study of such cases are to be placed in the scale against the results of careful physiological experiments, conducted on limited parts of the living, healthy brain!"

In conclusion, the reader said: "I am persuaded that such centres do exist, and I hazard the prediction, if it can be so called, that the time will soon come, when the supposition which admits such centres, will ripen into a certainty which none will question. While I speak thus, I am aware this doctrine is not clearly established, yet I expect to see not only the existence of such motor centres established, but, also, sense centres and perceptive centres, and centres for different kinds of memory and emotion, etc., and all in this wonderful nervous structure—the cortex of the brain."

The reader then extracted from a recent number of the *British Medical Journal* an account of Ferrier's latest experiments, which confirmed him in his prediction.

The paper was discussed by Drs. Bartlett, Merriman, Hyde, Owen, Starkweather, and others.

In behalf of the Society, the President invited Prof. Jewell to supplement this paper with another, at some future day, in which the practical issues of this important question should be treated.

Dr. Sawyer read a paper on the Intestinal Affections of Young Children.

---

### CHICAGO SOCIETY OF PHYSICIANS AND SURGEONS.

*Regular Meeting, Sept.* 27, 1875.

(Reported by E. WARREN SAWYER, M.D.)

